# Effect of Chitosan and Acrylic Acid Addition to Acrylic Resin on Porosity and *Streptococcus mutans* Growth in Denture Base

**DOI:** 10.1055/s-0042-1751002

**Published:** 2022-09-08

**Authors:** Titik Ismiyati, Ananto Ali Alhasyimi

**Affiliations:** 1 Department of Prosthodontics, Faculty of Dentistry, Universitas Gadjah Mada, Yogyakarta, Indonesia; 2 Department of Orthodontics, Faculty of Dentistry, Universitas Gadjah Mada, Yogyakarta, Indonesia

**Keywords:** chitosan, acrylic resin, denture base, porosity, *Streptococcus mutans*

## Abstract

**Objective** This work aimed to determine the effect of adding chitosan and acrylic acid to acrylic resin denture base on the porosity of the material and the growth of *Streptococcus mutans* .

**Materials and Methods** This study is an experimental laboratory research. Samples were divided into the following three groups ( *n* = 10): group 1 was the control group, group 2 was the acrylic resin mixture with 1% chitosan and acrylate acid, and group 3 was the acrylic resin mixture with 2% chitosan and acrylate acid. *S. mutans* growth was tested using the dilution method, and porosity was examined using an optical microscope. Data were calculated by one-way analysis of variance ( *p* < 0.05) and correlation analysis.

**Results** The acrylic resin added with 2% chitosan and acrylic acid showed pores with an almost spherical shape and the smallest size. Significant difference ( *p* < 0.05) was observed among all the groups. A positive and extremely strong correlation was found between porosity and *S. mutans* growth.

**Conclusion** Chitosan and acrylic acid at 1 and 2% concentrations can be added to acrylic resin to minimize the porosity of the denture base and reduce the growth of *S. mutans* . A less porous denture is associated with a low *S. mutans* growth rate.

## Introduction

As the older population develops, so does the need for dentures to restore stomatognathic function and improve the quality of life of patients. 1 Denture base made of polymethyl methacrylate acrylic resins' advantages include not just their acceptable aesthetic value, but also their relative ease of manipulation and low-cost availability. 2 An ideal base material can meet the mechanical, physical, and biological properties currently required for dentistry application. However, the drawback of acrylic resin is its porosity. 3 Pores in dentures can lead to high internal stresses and increase the susceptibility of the denture base to distortion and curvature, thus affecting its physical, aesthetic, and hygienic properties. 1 The porous surface of the denture base can provide opportunities for the attachment of microorganisms and the development of biofilms. 4 5 The oral cavity contains many microorganisms, and the microbial flora of the *Streptococcus mutans* is considered one of the most important cariogenic species. 6 7 *S. mutans* is the most common bacteria in plaque as its primary habitat and colonizes the surface of the teeth to form plaque. Denture plaque is the source of problems related to periodontal tissues, bad breath, denture discoloration, and pathogenic properties in the mouth and is the main cause of plaque, denture stomatitis, and caries. 8 *S. mutans* can grow depending on the media and the environment for bacterial growth. If the media and environmental conditions are suitable for these microorganisms, then they will grow in a relatively short time. These bacteria produce extracellular polysaccharides that adhere to a tooth enamel surface, denture base, or organic surface. 9 Using a mixed matrix with antimicrobial materials will endow the denture base with antibacterial properties. 10

Chitosan is a polysaccharide distilled from chitin that has specific bioactive, biocompatible, antibacterial, and biodegradable properties. 11 12 Therefore, chitosan shows potential as an antibacterial material due to its electrostatic interaction with bacteria. This compound has an amine functional group (NH _3_^+^ ) with a strong positive charge and binds to the negatively charged bacterial cell wall to change its permeability, thus resulting in bacterial death. In addition to functioning as an antibacterial, chitosan can also be used as an antifungal. Similar to its antibacterial function, its antifungal activity is mostly related to the interaction of positively charged polysaccharides with negatively charged residues on the fungal cell wall. 13 14 The functional groups in chitosan allow for various chemical modifications, such as reacting the cross-linking intermediates of chitosan with acrylic acid as a coupling agent to create cross-reactions that form new bonds. 15 16 Mixing polymethyl methacrylate and chitosan produces intermolecular and intramolecular bonds. Mixing acrylic resin with 2% chitosan and acrylic acid may produce an antifungal acrylic resin that can be used as a denture base material. 17 Furthermore, this study aimed to determine the effect of adding chitosan and acrylic acid at concentrations of 1 and 2% to acrylic resin on the porosity of the denture base and the development of *S. mutans* . The hypothesis for this study was that adding chitosan and acrylic acid to acrylic resin at concentrations of 1 and 2% would reduce the porosity of the denture base and inhibit the development of *S. mutans* .

## Material and Methods

### Material Preparation

This laboratory experiment (registration number No.00492/KKEP/FKG-UGM/EC/2020) was approved by the Research Ethics Commission of the Faculty of Dentistry, Universitas Gadjah Mada (UGM), Yogyakarta, Indonesia. Thirty research subjects were divided into three groups of 10. Group 1 was acrylic resin without a mixture (control), group 2 was a mixture of acrylic resin with 1% chitosan and acrylic acid, and group 3 was a mixture of acrylic resin with 2% chitosan and acrylic acid. The size of disc-shaped *S. mutans* specimens was 4 × 2 mm ^2^ , and that for porosity test was 20 × 20 × 2 mm ^3^ .

### Assessment of Porosity and *S. mutans* Growth

The porosity of the samples was determined by measuring the average porosity in each treatment group using an optical microscope at 100× magnification. The surface of each sample was divided into four viewing areas using a pencil, and the porosity in each area was calculated using a microscope. Total porosity was calculated using the formula: Total porosity = number of pores in all viewing areas divided by four. Furthermore, the size and geometry of the pores in each area and the whole perimeter could be observed after the scanned research subjects were displayed on the computer. With the help of software, the area of each pore was measured using the formula:

Pore diameter ( *d* ) was calculated according to the area parameter using the following equation 18 :

*d* = 2 √ *A* /π . Sphericity geometry was calculated using formula: 19 *Q* : 4 π *A* / *P* ^2^ ,

where Q is sphericity explanation, *d* is the diameter, A is the pore area, *P* is the perimeter, and π is constant (3.14).

*S. mutans* suspension was prepared according to the standard of 0.5 Farland (1.5 × 108 CFU/mL). The bacteria were obtained from the research laboratory of the Faculty of Dentistry, UGM. The research subjects were immersed in sterile distilled water for 48 hours, sterilized using autoclave for 18 minutes at 121°C, soaked in artificial saliva for 1 hour, and mixed to the suspension of *S. mutans* at 0.5 McFarland for 24 hours at 37°C. Each group was placed in a conical tube containing 10 mL of sterile distilled water for 8 hours, vibrated using a vortex, and diluted to 10 ^−3^ . A 0.01-mL sample was extracted, cultured for 48 hours in brain heart infusion agar in a petri dish and leveled using a speeder, incubated for 48 hours at 37°C, and finally counted using a colony counter.

### Statistical Analysis

A homogeneity test with Levene's test and normality test was carried out. Data were then statistically analyzed using one-way analysis of variance (ANOVA), followed by post hoc least significant different (LSD) test to examine significant differences between groups. Product–moment correlation was analyzed to determine the relationship between porosity and *S. mutans* growth, *p* < 0.05 was considered statistically significant. Analyses were carried out using the Statistical Package for the Social Sciences version 21 (IBM, USA).

## Results

**Fig. 1:**
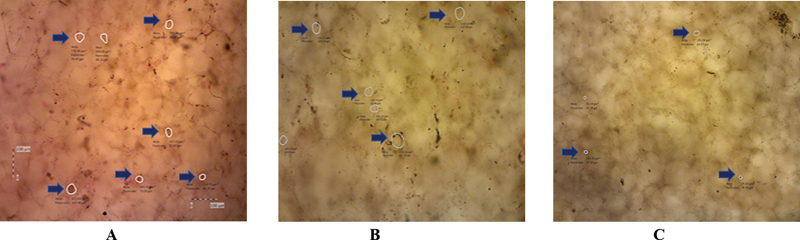
Pores in all group tested. ( **A** ) Pores in the control group. ( **B** ) Pores in the mixture of acrylic resin, chitosan, and acrylic acid 1%. ( **C** ) Pores in the mixture of acrylic resin, chitosan, and acrylic acid 2%.

Fig. 1A–C display the pores on groups 1 to 3, respectively. Pore characteristics are the most important factor in determining pore geometry and size, which was based on the area and perimeter. The perimeter was the length of the boundary line between each pore and the matrix, and the area was the pore area. Pore diameter ( *d* ) was determined on the basis of the area parameter. Table 1 lists the mean and standard deviation of the number and characteristics of pores.

**Table 1:** Descriptive value and analysis of variance comparisons of porosity characteristics

Parameter	G1	G2	G3	*p* -Value*	Post hoc comparison
Number of pores ( *n* )	107.00 ± 7.37	46.50 ± 14.08	13.67 ± 7.84	0.000 a	G1 > G2 > G3
Diameter of pores (µm)	29.02 ± 5.82	20.92 ± 5.92	11.34 ± 3.44	0.001 a	G1 > G2 > G3
Geometry of pores (o)	0.95 ± 0.18	0.94 ± 0.10	0.97 ± 0.06	0.923 b	NS b

Note: Group 1: Acrylic resin without mixture. Group 2: Acrylic resin with chitosan and acrylic acid at a concentration of 1%. Group 3: Acrylic resin with chitosan and acrylic acid at a concentration of 2%. Values are presented as mean ± standard deviation or *p* -Value only.

*By analysis of variance (ANOVA).

a Significant differences between groups ( *p* < 0.05).

b No significant (NS) differences between groups ( *p* > 0.05).

According to the analysis, each group showed a decreased average number of pores after being added with chitosan and acrylic acid at 1 and 2% concentrations. The highest value was found in group 1, followed by group 2, and the lowest was observed for group 3. The data were homogeneous (0.342 > 0.05) and normally distributed (0.867; 0.562; 0.347 > 0.05), and one-way ANOVA indicated significant difference among the groups ( *p* < 0.05). In addition, post hoc LSD test showed significant difference in average porosity between the no treatment group 1 (control) and treatment group 2 (1% concentration), between the no treatment (control) and treatment group 3 (2% concentration), and between the treatment groups 2 and 3 ( *p* < 0.05).

In terms of mean pore diameter, the largest value was observed for group 1 followed by groups 2 and 3. Homogeneity was calculated as 0.189 > 0.05, indicating that the data were homogeneous and normally distributed (0.744; 0.531; 0.101 > 0.05). One-way ANOVA showed significant differences among all groups ( *p* < 0.05). In addition, post hoc test showed significant differences among the three groups ( *p* < 0.05).

In terms of geometry, group 1 had the smallest average, and group 3 showed the largest average. Homogeneity value was 0.106 > 0.05, indicating that the data were homogeneous. Normality value of the distribution was 0.324, 0.42, and 0.109 > 0.05, implying that the data were normally distributed. ANOVA showed the lack of significant difference in pore geometry among all groups (0.923 > 0.05) with a mean value close to 1 in the range of 0.94 to 0.97 degree.

Table 2 lists the mean and standard deviation values for *S. mutans* growth in the three groups. The control group had the highest mean value, which then decreased in the treatment groups 2 and 3. Shapiro–Wilk normality test showed that group 1 had a score of 0.757, group 2 had a score of 0.563, and group 3 had a score of 0.892. The obtained *p* -value was > 0.05, indicating that the variable of *S. mutans* growth was normally distributed. Levene's homogeneity test showed that the growth variable of *S. mutans* had a *p* -value (significant) of 0.877, indicating that this variable was homogeneous.

**Table 2:** Mean and standard deviation of *Streptococcus mutans* (10 ^–^^3^ CFU/mL) on acrylic resin and the mixture of acrylic resin with chitosan and acrylic acid at concentrations of 1 and 2%

Group	*n*	*S. mutans* (10 ^−3^ CFU/mL)	*p* -Value	Post hoc		
				Group 1	Group 2	Group 3
Group 1	10	291.50 ± 10.93	0.000 a	0.001 a	0.001 a	0.000 a
Group 2	10	173.00 ± 15.90				0.000 a
Group 3	10	106.67 ± 13.04				

Note: Group 1: Acrylic resin without mixture. Group 2: Acrylic resin with chitosan and acrylic acid at a concentration of 1%. Group 3: Acrylic resin with chitosan and acrylic acid at a concentration of 2%. Values are presented as mean ± standard deviation or *p* -Value only.

a By analysis of variance (ANOVA), significant differences between groups ( *p* <0.05).

One-way ANOVA revealed that *S. mutans* growth had a *p* -value of 0.00, implying a significant difference between the control and treatment groups. Post hoc LSD test showed significant difference in the mean value of *S. mutans* growth between groups 1 and 2, between groups 1 and 3, and between groups 2 and 3.

**Fig. 2:**
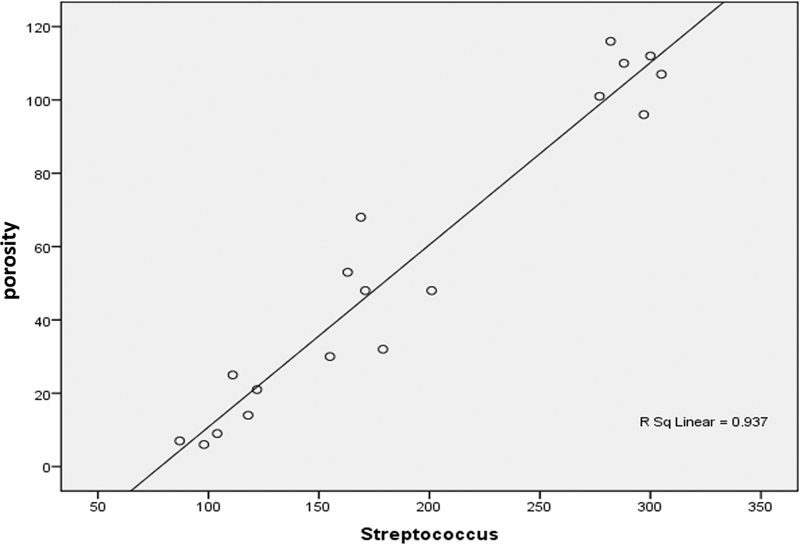
Correlation graph between *Streptococcus mutans* growth and chitosan–acrylic denture base.

Product–moment correlation analysis showed that the correlation coefficient between porosity and *S. mutans* growth was 0.968 with a *p* -value of < 0.05, indicating a significant positive and extremely strong correlation between these variables. Fig. 2 shows the correlation graph between the variables of porosity and *S. mutans* growth.

## Discussion

Porosity in acrylic resin is often regarded as an undesirable characteristic for denture bases because of its effect on the physical, aesthetic, and mechanical properties. 1 19 Dentures in the mouth are always in contact with various oral microorganisms such as *Candida albicans* . 8 20 Therefore, the porosity and *S. mutans* growth in acrylic resin must be reduced by adding other ingredients, such as chitosan with known antibacterial and antifungal properties. In this study, the acrylic resin without mixture (control) had the largest average number of pores compared with the acrylic resin mixed with chitosan and acrylic acid at 1 and 2% concentrations. This phenomenon occurred because even after the polymerization of acrylic resin without mixture, some monomers did not bind to the polymer and instead form pores. Meanwhile, the acrylic acid can function as a coupling agent to form bonds between acrylic resin and chitosan; hence, the monomers contained in the acrylic resin can be bonded during polymerization. This finding was confirmed by the reducing amount residual monomer in the acrylic resin mixtures with 1 and 2% chitosan and acrylic acid. 21 Therefore, this polymerization can reduce the formation of pores. The decrease in the amount of residual monomer can reduce the formation of empty space known as porosity.

Another possibility is that the formation of porosity can be caused by the lack of agitation during mixing. This situation is reinforced by the fact that porosity can be minimized by ensuring the homogeneity of the mixture. In addition, the quality of the polymer and monomer mixture depends on various parameters, such as mixing temperature and pressing pressure. In the production of acrylic resin with best quality, the effect of these parameters on porosity must be considered because a large number of pores leads to a small volume of acrylic resin. The porosity of acrylic resin depends on several complex factors, including formulation and polymerization method. Pores can also cause distortion, leading to a decrease in the strength of the denture. 22 Pore diameter can be analyzed using the area and perimeter of the images taken with the image software. The average pore diameter of acrylic resin without mixture was larger than that of the acrylic resin with 1 and 2% chitosan and acrylic acid. In this work, the average diameter of the acrylic resin was 11.34 to 29.02 µm. Another study found that the diameter of acrylic resin was between 35 and 267µm. 17 Although the pore diameter in the present work was smaller than previous values, the American Dental Association stated that no bubbles or pores must be present in polymer denture bases when viewed directly without a microscope. A large pore size will allow the easy absorption of liquid, which in turn will reduce the density and other mechanical properties of the acrylic resin. 22 23 In addition to the small size, the pore shapes in all groups were almost spherical (0.94–0.7 degree). This spherical pore geometry is assumed to have superior mechanical strength. 24

The growth of *S. mutans* on the acrylic resin without mixture was greater than that on the acrylic resin mixed with chitosan and acrylic acid. The acrylic resin without the mixture did not exhibit an antibacterial activity. Meanwhile, for the acrylic resin mixed with chitosan and acrylic acid, the *S. mutans* growth decreased with the increasing concentration of chitosan and acrylic acid in acrylic resin. Chitosan's antibacterial activity is most commonly attributed to its binding to the negatively charged bacterial cell wall, thereby causing cell breakdown and affecting membrane permeability; in addition, chitosan binds to the bacterial deoxyribonucleic acid (DNA), thus inhibiting DNA replication and ultimately leading to cell death. 25 26 As a chelating agent, chitosan may also induce toxins by binding to trace metal elements and preventing microbiological development. 27 At high chitosan concentrations, microorganisms either die or their growth is blocked. This finding is in accordance with a previous study on the effects of a mixture of chitosan and acrylic on *C. albicans* . 16 The current results are also in agreement with Heryumani Sulandjari et al, who found that toothpaste containing chitosan can reduce plaque accumulation. 28

Significant difference in porosity and *S. mutans* growth was observed among all the groups. Bacterial replication probably did not occur because chitosan contains compounds that can affect permeability and cause an imbalance in bacterial cells. 29 Chitosan has a single primary amine group and, as a result, is obviously a cationic biomaterial due to the presence of a free NH _3_^+^ . Cationic compounds exert their antibacterial action by destroying the structure and function of bacteria's cell wall and membrane. Bacterial cells are surrounded by a peptidoglycan wall comprised of N-acetylglucosamine, N-acetylmuramic acid, and D and L amino acids that bind the positively charged amine groups of chitosan oligomers to glycine in the peptidoglycan structure. As a result, the cell wall is disrupted, exposing the cell membrane to osmotic shock. As a result, the cytoplasmic contents are ejected and the cell dies. 14 The positive correlation coefficient indicated that the correlation between the two variables was unidirectional, that is, the higher the porosity variable, the higher the *S. mutans* variable, and vice versa. The basic difference between acrylic resin and acrylic resin mixed with chitosan was their number of pores. The acrylic resin mixed with chitosan and acrylic acid had a low porosity, which hinders bacterial growth. By contrast, the acrylic resin without the mixture had a high porosity, which is suitable for *S. mutans* growth. One weakness of this study was that it was conducted *in vitro* , and so did not reflect the whole range of oral settings. The mechanical forces and strains experienced in the oral cavity are distinct from those experienced in the laboratory, when specimens are subjected to each condition individually. Future research must be conducted in a manner that more closely resembles the *in vivo* environment in order to provide more significant results.

## Conclusion

Chitosan and acrylic acid at 1 and 2% concentrations can reduce the porosity of acrylic resins and limit the growth of *S. mutans* . The acrylic resin with chitosan and acrylic acid had pores with an almost spherical shape and the smallest size. A less porous material is associated with a low *S. mutans* growth rate.
